# Making the investment case for national regulatory authorities

**DOI:** 10.1186/s40545-021-00299-7

**Published:** 2021-01-21

**Authors:** Gloria Twesigye, Tamara Hafner, Javier Guzman

**Affiliations:** USAID Medicines, Technologies, and Pharmaceutical Services (MTaPS) Program, Management Sciences for Health, Arlington, VA USA

**Keywords:** Regulatory systems, National regulatory authorities, Health systems strengthening, Access to medicines, Quality-assured medicines

## Abstract

Well-functioning national regulatory authorities (NRAs) ensure access to safe, effective, quality-assured, and affordable medical products. However, the benefits of their work are often unseen and difficult to attribute, thereby making NRAs undervalued and under-resourced, particularly in low- and middle-income countries. This paper offers three key arguments NRAs and other stakeholders can use to advocate for greater investment in regulatory systems strengthening—medical products regulation effectively safeguards public health; effective regulation improves health system’s efficiency by increasing access to affordable medical products, contributing to universal health coverage; and robust regulation strengthens local pharmaceutical manufacturing and bolsters pharmaceutical trade. NRAs’ critical role in health systems is indisputable, yet they need to better promote their value to receive the requisite resources to function effectively.

## Introduction

As part of its proposed strategy for strengthening regulatory systems to support good quality, wholesome food and safe, effective medical products globally, the National Academies of Sciences, Engineering, and Medicine (NASEM) recommended that national regulatory agencies (NRAs) should address the “ways regulation improves quality, safety, and access, using different strategies to convey this information to government leaders, regulated industry, and the public” [1]. NRAs tend to be in the spotlight during highly visible public health crises. The benefits of their work, when done well, are diffused, difficult to attribute, and hidden from the public [1].

The World Health Organization (WHO) estimates that only 30% of NRAs have the capacity to effectively and efficiently regulate medical products in their countries and that one-third of the world’s population lacks timely access to quality-assured medicines [2]. Systems strengthening is resource-intensive and requires a long time commitment [3]. Further, the evidence base on what systems strengthening interventions are effective has been historically weak [3]. With the development of the Global Benchmarking Tool (GBT) Revision VI, the first globally accepted tool for objectively assessing and strengthening NRAs, countries now have a tool to formulate an institutional development plan with realistic standards and well-defined interventions to systematically strengthen their system [4]. The availability of a globally agreed-upon benchmark for regulatory systems underscores the need for increased national-level commitments to strengthening NRAs [3].

Prompted by the NASEM’s recommendation for better communication, we propose three arguments for greater investment in regulatory systems strengthening. These arguments are particularly salient in low- and middle-income countries (LMICs) where many NRAs are chronically underfunded and lack the necessary legal mandate and resources to effectively control the safety, efficacy, and quality of medical products being imported, manufactured, or used in their jurisdictions [1]. Weak NRAs also affect the affordability of medical products, as they influence market competitiveness, and the availability of substandard and falsified medicines and underuse of generic medicines, two of the highest sources of inefficient health spending [5]. NRAs’ inability to conduct core regulatory functions negatively affects population health and wellbeing, the efficiency and sustainability of health systems, and the robustness of local pharmaceutical industry and trade.

## Governments should regulate medical products effectively to safeguard public health

Regulation is indisputably a public health good because it ensures access to safe, quality-assured, and affordable medical products. A 2018 meta-analysis revealed that 13.6% of essential medicines in LMICs were substandard and falsified, including 18.7% in Africa and 13.7% in Asia [6]. Models estimate poor-quality medicines cause approximately 70,000 excess deaths from childhood pneumonia and 8500 to 20,000 malaria deaths in sub-Saharan Africa annually [7]. Weak NRAs have limited capacity to detect and prevent the sale and consumption of substandard and falsified medicines. This contributes to poor health outcomes by prolonging disease, increasing mortality and adverse events, and hastening antimicrobial resistance [7]. Without a functional NRA, a government cannot verify whether the medical products being imported or manufactured locally meet approved quality standards or that those standards are stringent enough to protect consumers. Likewise, the public cannot be confident that the products they use are safe and effective and may lose confidence in both the health system and the government [1].

The public health argument has been strengthened by the COVID-19 pandemic as NRAs are essential in addressing the unique challenges associated with deploying new medical products during a public health emergency. With new vaccines and medicines, oversight of clinical trials is crucial to ensure they are appropriately designed and patients’ rights and safety are protected. Similarly, the expedited registration of new products—including medical devices, diagnostics, and personal protective equipment—must guarantee product safety, efficacy, and quality assurance. As these new products enter the market, vigilance to detect and address adverse events will be equally critical.

## Effective regulation improves health systems’ efficiency, increases access to medical products, and contributes to achieving universal health coverage

The significant economic costs of substandard and falsified medicines directly hamper progress towards universal health coverage (UHC), a target of Sustainable Development Goal (SDG) 3 [5]. WHO estimates that expenditures on falsified and substandard medicines in LMICs are approximately US$ 30 billion [7]. Importantly, patients bear the brunt of the economic costs through increased out-of-pocket expenses to pay for health services and medical products, as well as forgone earnings and lost productivity due to prolonged illness. Reducing the proportion of large household expenditures on health as a share of total household expenditures is a key indicator in measuring progress towards UHC (SDG Indicator 3.8.2); however, progress will be limited unless NRAs can reduce the availability of falsified and substandard medicines [8].

In many LMICs, prices for generic and brand medicines are often higher relative to high-income countries, and quality-assured generics remain unavailable and underutilized [5, 9]. Prioritizing the entry and use of generic medicines can have considerable cost savings. Strong regulatory systems allow for a fair and competitive market, removing low-priced, substandard products from the market. Cameron and Liang found that 17 countries could reduce expenditures for 18 medicines by an average of 60% solely by switching to generic medicines, saving an average of US$ 31.3 million in 1 year [9]. In Mexico, reforms initiated in 2011 to strengthen the Comisión Federal para la Protección contra Riesgos Sanitarios (COFEPRIS), the regulatory body, resulted in a 77% increase in the use of generic medicines over 2 years [10]. Increasing market penetration of quality-assured generic medicines while removing substandard products further enables the inclusion of an affordable basket of quality-assured essential medicines in health insurance schemes, promoting equitable access to medicines [11].

## Robust regulation strengthens local manufacturing and bolsters pharmaceutical trade

Effective regulation improves the quality of locally manufactured products and facilitates entry in international markets, thereby strengthening the local pharmaceutical industry and boosting trade. Patients and governments also benefit from greater competition and lower prices. Manufacturers from countries with strong regulatory authorities often receive preferential treatment in regional markets. After the COFEPRIS reforms, for example, PAHO recognized COFEPRIS as a reference authority, expanding the export market and the potential for expedited assessments in the Americas [10]. Additionally, strengthening COFEPRIS eliminated a backlog of approximately 4500 applications and led to an estimated 13.2% growth in the local Mexican pharmaceutical market between 2011 and 2014 [10]. El Salvador’s 2012 regulatory system reforms reduced case backlogs and increased competition in the market and the availability of quality-assured generics, contributing to an average 20–25% reduction in medicines prices and an approximate US$ 60 million annual savings in out-of-pocket medicines expenditures [12].

As NRAs mature and participate in regional harmonization and convergence initiatives, they increasingly collaborate to systematically rely on decisions and actions of NRAs recognized as reference authorities. For example, joint dossier reviews through regional regulatory harmonization initiatives shortened timelines for medicines registration for countries in the East African Community and the Southern African Development Community [13]. In the Caribbean Community, abbreviated dossier reviews helped streamline the process for generics through reliance on reference authorities [13]. Increased efficiencies in the medicines registration process reduce delays and costs to manufacturers, increase trade opportunities, and allow NRAs to redirect their limited resources to other essential regulatory functions, such as vigilance.

## Conclusion

Given NRAs’ indisputable, though largely invisible, role in national health systems and pharmaceutical markets, it is critical for NRAs to communicate the health and economic benefits of their work to the government, industry and the public. However, analyzing the health and economic impact of regulatory policies is challenging because of the time lag and indirect causal pathway. Some NRAs’ inability to generate evidence about the regulatory environment compounds the challenge. An implicit issue raised by this paper is the need for more systematic analyses of the health and economic benefits of medical products regulation, particularly in LMICs. Regardless, the COVID-19 pandemic has brought into sharp relief the need to invest in regulatory systems strengthening to ensure timely access to safe, effective, quality-assured, and affordable medical products. A strong regulatory system helps facilitate a robust response to pandemics and other health emergencies, as opposed to initiating an emergency response without the requisite systems in place.

## Data Availability

Not applicable.

## Abbreviations

COFEPRIS: Comisión Federal para la Protección contra Riesgos Sanitarios
GBT: Global Benchmarking Tool
LMIC: Low- and middle-income country
NASEM: National Academies of Sciences, Engineering, and Medicine
NRA: National Regulatory Authority
SDG: Sustainable Development Goal
UHC: Universal health coverage
WHO: World Health Organization
